# Association between psoriasis and peripheral artery occlusive disease: a population-based retrospective cohort study

**DOI:** 10.3389/fcvm.2023.1136540

**Published:** 2023-06-12

**Authors:** Chao-Bin Yeh, Liang-Tsai Yeh, Shun-Fa Yang, Bo-Yuan Wang, Yu-Hsun Wang, Chi-Ho Chan

**Affiliations:** ^1^ Department of Emergency Medicine, School of Medicine, Chung Shan Medical University, Taichung, Taiwan; ^2^ Department of Emergency Medicine, Chung Shan Medical University Hospital, Taichung, Taiwan; ^3^ Institute of Medicine, Chung Shan Medical University, Taichung, Taiwan; ^4^ Department of Anesthesiology, Changhua Christian Hospital, Changhua, Taiwan; ^5^ Department of Post-Baccalaureate Medicine, College of Medicine, National Chung Hsing University, Taichung, Taiwan; ^6^ Department of Medical Research, Chung Shan Medical University Hospital, Taichung, Taiwan; ^7^ Department of Microbiology and Immunology, Chung Shan Medical University, Taichung, Taiwan

**Keywords:** psoriasis, peripheral arterial occlusive diseases, risks, retrospective cohort study, propensity score matching

## Abstract

**Introduction:**

Psoriasis (PSO) is a chronic skin condition that affects a variety of disorders, especially the cardiovascular system. This study investigated the association between PSO and peripheral arterial disease (PAOD).

**Methods:**

A retrospective cohort study design was carried out between 2000 and 2018. The exposure subject was a newly diagnosed PSO. The diagnosis of PSO was never elaborated as a comparison subject. Balanced heterogeneity of the two groups was used by propensity score matching. The cumulative incidence of PAOD between the two groups was performed using Kaplan-Meier analysis. The Cox proportional hazard model was used to measure the risk of PAOD risk hazard ratio.

**Results:**

After matching the 1: 1 propensity score, 15,696 subjects with PSO and the same number of subjects without the diagnosis of PSO were recruited. The PSO subject had a higher risk of PAOD than the non-PSO subject (adjusted HR = 1.25; 95% CI = 1.03-1.50). In the 40-64-year-old subgroup, the subject of PSO exhibited an increased risk of PAOD than the subject without PSO.

**Conclusion:**

Psoriasis is associated with an increased risk of peripheral arterial disease and curative care is necessary to reduce the risk of PAOD..

## Introduction

Psoriasis (PSO) is a chronic, recurrent, inflammatory disease. More than 125 million people, approximately 1%–2% of the general population worldwide, had PSO in 2020 (1, 2). However, the prevalence of PSO differs geographically. Generally, the incidence rate of PSO in Asian countries is less than in western countries. A survey in six cities in China showed a prevalence of PSO of only 0.47% (3). Although no specific survey has been conducted on the prevalence of PSO in Taiwan, a study using the National Health Insurance Research Database identified 53,761 PSO patients from a population of 23 million in 2011 (4). Therefore, the prevalence of PSO is approximately 0.24% in Taiwan. Based on its appearance in clinical presentations, PSO is classified into different forms, psoriasis vulgaris, inverse psoriasis, guttate psoriasis, pustular psoriasis, and erythrodermic psoriasis, while psoriasis vulgaris is a common form (1, 5).

Evidence showed that PSO can contribute to cardiovascular risk. For example, chronic inflammation in PSO patients increased the accumulation of visceral adipose tissue and the noncalcified burden of the coronary artery (6). PSO patients had a higher level of C-reactive protein, chemerin, fetuin-A, and osteopontin in the blood, which are predictors of cardiovascular risk (7). In addition, PSO can also promote severe vascular events such as myocardial infarction, stroke, and cardiovascular mortality (8). A higher portion of patients with PSO was found to have arterial stiffness compared to non-PSO patients (9). PSO can contribute to hypercoagulability in blood vessels to form thrombus (10).

Peripheral arterial occlusive disease (PAOD) is an atherosclerosis that usually occurs in the lower limb artery with the formation of arterial thrombosis or embolism. Initially, PAOD is not easily recognized unless intermittent claudication is evident (11). Severe PAOD can cause atherosclerotic stenosis, loss of limb, or even death (12). Ankle–brachial arterial index test, computed tomography, and magnetic resonance imaging were generally applied for the early detection of PAOD (13, 14).

Not only can PAOD develop into serious diseases, but it also increases the risk of developing other cardiovascular diseases (CVDs), such as coronary heart disease and myocardial infarction (MI) (15, 16). PSO is also associated with the risk of certain CVD, i.e., MI, hypertension, arrhythmia, and valvular disease (17, 18, 19).

Pathological changes in PSO and PAOD have certain common mechanisms based on the immune response. For example, significantly higher serum levels of cytokines such as tumor necrosis factor-alpha (TNF-α), interleukin 4 (IL-4), and interleukin 6 (IL-6) are observed in both diseases (20, 21). A β-galactoside-binding lectin, galectin-3, which is associated with cell proliferation, inflammation, and angiogenesis, plays a role in both PSO and PAOD (22, 23, 24).

Considering the increased risk of CVD and common immunological markers for PSO and PAOD, we enrolled both patients with and without PSO from the 2000 Longitudinal Health Insurance Database (LHID 2000) and investigated whether PSO influences the risk of PAOD. We hypothesized that patients with PSO would have a significantly higher risk of PAOD.

## Materials and methods

### Data sources

We retrieved a longitudinal health insurance database that was managed by the Health and Welfare Data Center (HWDC) of Taiwan. There are 2 million random sampling subjects from the registry of beneficiaries in the whole population for the year 2000. The database included data on outpatient, emergency, and hospitalization medical records, including medication, surgical operation, procedure, and expenditure data from 2000 to 2018. This study was certified by the institutional review board of Chung Shan Medical University Hospital (CS1-20056).

### Study group and outcome

We conducted a retrospective cohort study. The study included individuals who were newly diagnosed with PSO (ICD-CM codes: 696.1, 696.2, 696.3, 696.4, 696.5, L30.5, L40.0–L40.4, L40.8, L40.9, L41.0, L41.1, L41.3–L41.5, L41.8, L41.9, L42, L44.0, L94.5) at onset time from 2002 to 2017. To ensure accuracy, patients were included if any only if their diagnosis was given by a rheumatologist or dermatologist for ≥3 times outpatient visits or ≥1 inpatient stay. The index date was defined as the date of initial diagnosed PSO. In order to ensure that all instances of PAOD would be new onset PAOD, patients with a diagnosis of PAOD before the index date were excluded. The non-PSO group was defined as never diagnosed psoriasis and similar disorders (ICD-CM codes: 696, L40, L41, L42, L44, L45, L30.5, L94.5) from 2000 to 2018.

The primary outcome was a diagnosis of PAOD (ICD-CM codes: 443.8, 443.9, 444, I73.81, I73.89, I73.9, I74.01, I74.09, I74.11, I74.2, I74.3, I74.4, I74.5, I74.8, I74.9, I79.1, I79.8). All samples were traced until the outcome of interest, death, or the end of 2018, whichever came first.

### Covariates and matching

The demographic characteristics included in the analysis were age and sex and the comorbidities of hypertension (ICD-CM codes: 401–405, I10–I15), hyperlipidemia (ICD-CM codes: 272, E78), chronic liver disease (ICD-CM codes: 571, K70, K73, K74, K75.4, K75.81, K76.0, K76.89, K76.9), chronic kidney disease (ICD-CM codes: 585, N184, N185, N186, N189), diabetes (ICD-CM codes: 250, E10, E11, E13), chronic obstructive pulmonary disease (ICD-CM codes: 491, 492, 496, J41–J44), ischemic heart disease (ICD-CM codes: 410–414, I20–I25), stroke (ICD-CM codes: 433–438, I63, I64), and intracranial bleeding (ICD-CM codes: 430–432, I60–I62). An individual was defined as having these comorbidities if and only if they received a diagnosed within 1 year before the index date for ≥3 outpatient visits or ≥1 inpatient stay.

First, a 1:4 age and sex matching was performed to ensure an identical index date for any given PSO participant with their non-PSO counterparts. If the non-PSO group had a previous diagnosis of PAOD when matched to the corresponding index date, that non-PSO group would be excluded and rematched with a new comparison group. Subsequently, the propensity score matching (PSM) was carried out by age, sex, and comorbidities, including the presence of hypertension, hyperlipidemia, chronic liver disease, chronic kidney disease, diabetes, chronic obstructive pulmonary disease, ischemic heart disease, stroke, and intracranial bleeding comorbidities. The propensity score was calculated using a binary logistic regression. The binary variable was the diagnosis of PAOD. PSM ensured the balanced heterogeneity of the two groups.

### Statistical analysis

The comparison of the PSO and non-PSO groups was performed using the absolute standardized difference (ASD). An ASD <0.1 indicated similarity in the characteristics of both groups (25). A Poisson regression model and Kaplan–Meier analysis were used to measure the relative risk (RR) and the cumulative incidence of PAOD between the two groups. To measure independent risk factors for PAOD, hazard ratios were evaluated using a Cox proportional hazards model. Moreover, sensitivity analysis was conducted in this study to reduce potential confounding factors by controlling for medications, including antiplatelet, anticoagulant, methotrexate, sulfasalazine, and corticosteroids. The statistical software was SAS version 9.4 (SAS Institute, NC, USA).

## Results

### Demographic characteristics

**Figure 1:**
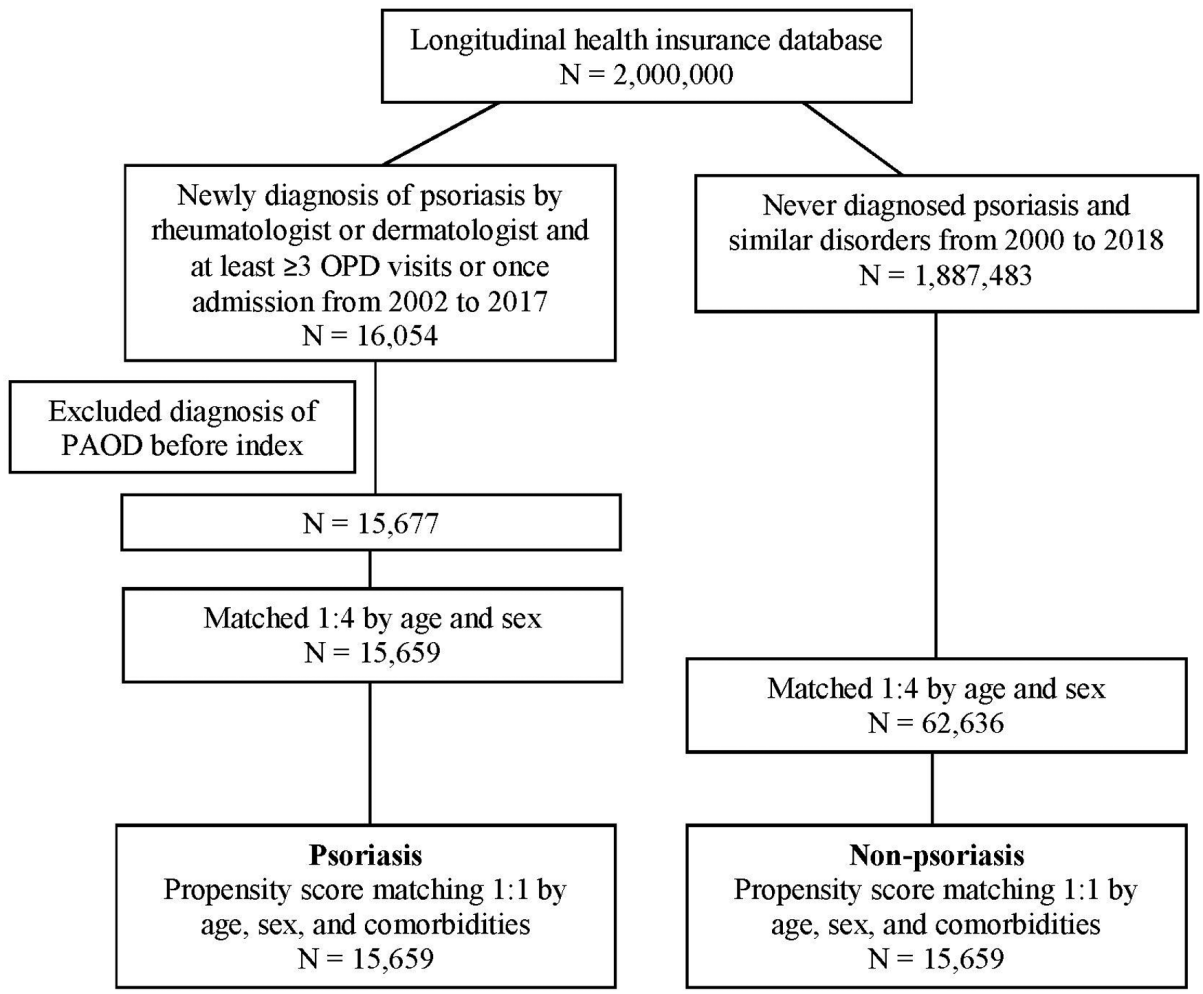
Flowchart of patient selection.

In total, 16,054 patients with PSO and 1,887,483 patients who had never been diagnosed with PSO were recruited. After excluding patients with a diagnosis of PAOD before the index date, 15,677 patients were in the PSO cohort. Subsequently, 1:1 PSM by age, sex, and comorbidity was analyzed. Finally, 15,659 patients in the PSO cohort and 15,659 patients without PSO in the matched cohort were analyzed for their risk of PAOD (Figure 1). The mean age of patients with and without PSO was 39.30 ± 19.26 and 39.51 ± 19.32, respectively. The male/female ratio was similar in both groups (55.7%:44.3% in the non-PSO group; 55.6%:44.4% in the PSO group). Before PSM, the PSO group compared with non-PSO group had slightly higher proportion in hypertension, hyperlipidemia, chronic liver disease, chronic kidney disease, diabetes, COPD, ischemic heart disease, stroke, and intracranial bleeding. After PSM, all ASD were <0.1. It represented that age, sex, and comorbidities were similar between both groups (Table 1).

**Table 1:** Demographic characteristics of PSO and non-PSO.

	Before PSM matching					After PSM matching				
	Non-PSO (*N* = 62,636)		PSO (*N* = 15,659)			Non-PSO (*N* = 15,659)		PSO (*N* = 15,659)		
** **	*n*	%	*n*	%	ASD	*n*	%	*n*	%	ASD
Age					<0.001					<0.001
<20	10,272	16.4	2,568	16.4		2,571	16.4	2,568	16.4	
20–39	23,676	37.8	5,919	37.8		5,912	37.8	5,919	37.8	
40–64	21,264	33.9	5,316	33.9		5,313	33.9	5,316	33.9	
≥65	7,424	11.9	1,856	11.9		1,863	11.9	1,856	11.9	
Mean ± SD	39.30 ± 19.26		39.30 ± 19.26		<0.001	39.51 ± 19.32		39.30 ± 19.26		0.011
Age					<0.001					0.001
<65	55,212	88.1	13,803	88.1		13,796	88.1	13,803	88.1	
≥65	7,424	11.9	1,856	11.9		1,863	11.9	1,856	11.9	
Sex					<0.001					0.002
Female	27,832	44.4	6,958	44.4		6,942	44.3	6,958	44.4	
Male	34,804	55.6	8,701	55.6		8,717	55.7	8,701	55.6	
Hypertension	6,999	11.2	2,138	13.7	0.075	2,017	12.9	2,007	12.8	0.002
Hyperlipidemia	3,556	5.7	1,133	7.2	0.063	949	6.1	947	6.0	0.001
Chronic liver disease	1,818	2.9	670	4.3	0.074	491	3.1	499	3.2	0.003
Chronic kidney disease	409	0.7	130	0.8	0.021	120	0.8	119	0.8	0.001
Diabetes	3,270	5.2	1,048	6.7	0.062	983	6.3	981	6.3	0.001
COPD	1,037	1.7	348	2.2	0.041	258	1.6	253	1.6	0.003
Ischemic heart disease	1,936	3.1	583	3.7	0.035	522	3.3	512	3.3	0.004
Stroke	1,131	1.8	363	2.3	0.036	331	2.1	330	2.1	<0.001
Intracranial bleeding	150	0.2	49	0.3	0.014	34	0.2	43	0.3	0.012

COPD, chronic obstructive pulmonary disease; PSO: psoriasis.

### Incidence of PAOD

**Figure 2:**
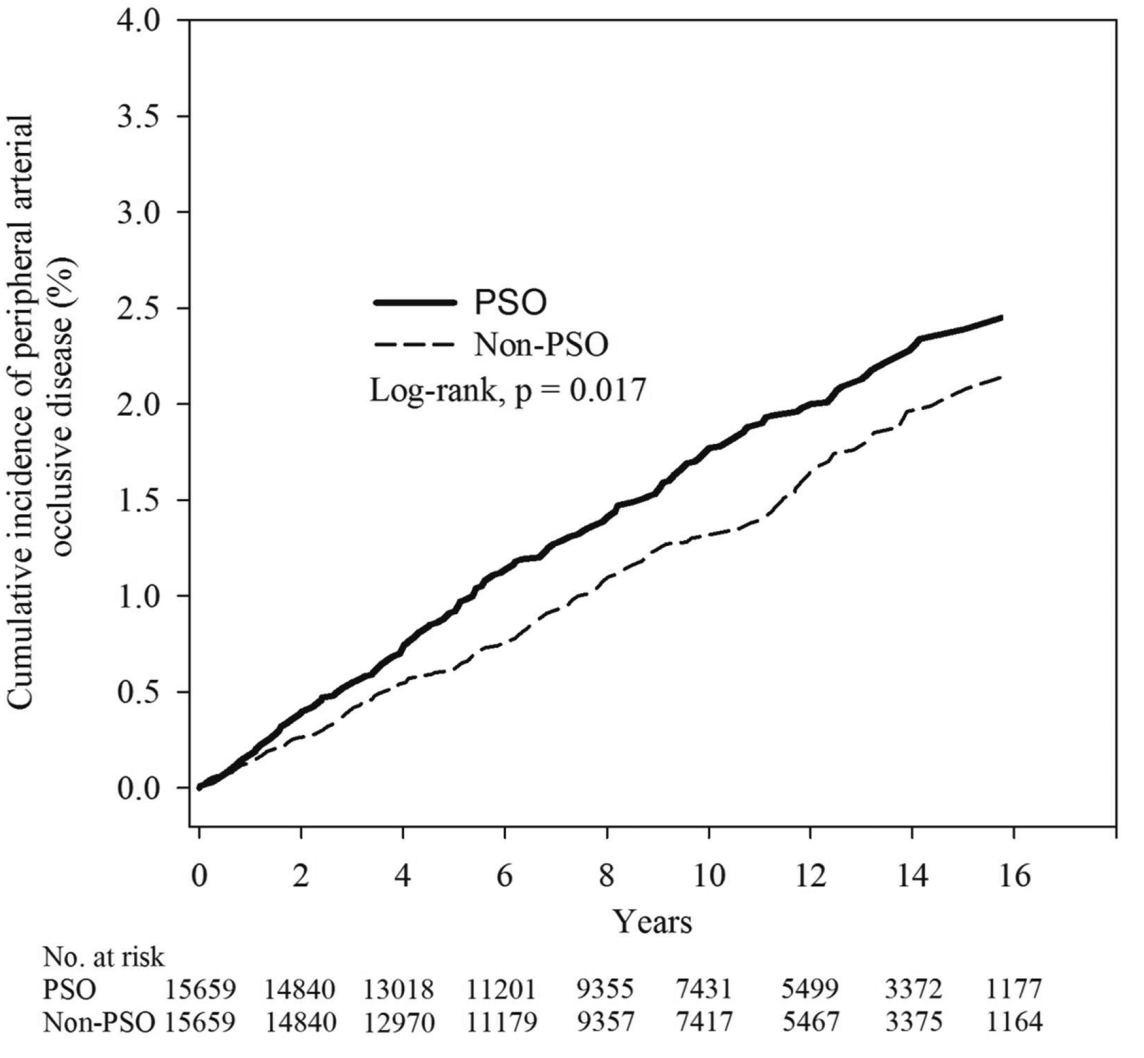
Kaplan–Meier curves of the cumulative proportions of PAOD in psoriasis and non- psoriasis patients.

Subsequently, we used Poisson regression to compare the relative risk of PAOD between the two groups. The incidence density of the PSO group and the non-PSO group was 1.69 (95% C.I. = 1.49–1.92) and 1.35 (95% C.I. = 1.17–1.55), respectively. The relative risk of developing PAOD in PSO group compared to non-PSO group was 1.26 (95% C.I. = 1.04–1.51) (Table 2). The mean time of developing PAOD with and without PSO was 5.36 and 5.91 years. The log-rank test (*p* = 0.017) showed that the cumulative incidence of PAOD was higher in the PSO group than in the non-PSO group (Figure 2).

**Table 2:** Poisson regression of relative risk of PSO and non-PSO.

	Non-PSO	PSO
*N*	15,659	15,659
Person-years	1,46,897	1,47,186
No. of PAOD	198	249
ID (95% C.I.)	1.35 (1.17–1.55)	1.69 (1.49–1.92)
Relative risk (95% C.I.)	Reference	1.26 (1.04–1.51)

ID, Incidence density (per 1,000 person-years); PSO, psoriasis; PAOD, peripheral arterial disease.

### Risk of PAOD between PSO and non-PSO groups

The PSO group had an increased risk of PAOD (adjusted HR = 1.25; 95% C.I. = 1.03–1.50). Patients aged 20 to 39 years (adjusted HR = 3.87 years; 95% C.I. = 1.65–9.07 years), 40 to 64 years (adjusted HR = 18.37 years; 95% C.I. = 8.13–41.49 years) and ≥65 years (adjusted HR = 36.70 years; 95% C.I. = 16.00–84.17 years) had a higher risk of PAOD. Men had an increasing risk of PAOD than women (adjusted HR = 1.24; 95% C.I. = 1.02–1.51). Furthermore, comorbidities such as hypertension, chronic kidney disease, diabetes, and stroke were also risk factors for PAOD (Table 3). In the 40 to 64-year-old subgroup, PSO had a significant interaction with PAOD risk in a subgroup analysis (adjusted HR = 1.34; 95% C.I. = 1.03–1.75; *p* for interaction < 0.001; Table 4). In a sensitivity analysis, after controlling for methotrexate, corticosteroids, anticoagulants, and antiplatelet agents, the PSO group had an increased risk of PAOD (adjusted HR = 1.52; 95% C.I. = 1.23–1.88) (Supplementary Table S1).

**Table 3:** Cox proportional hazard model analysis for risk of PAOD.

	Univariable	*p* value	Multivariable^†^	*p* value
	HR (95% C.I.)		HR (95% C.I.)	
Group				
Non-psoriasis	Reference		Reference	
Psoriasis	1.26 (1.04–1.51)	0.017	1.25 (1.03–1.50)	0.021
Age				
<20	Reference		Reference	
20–39	3.94 (1.68–9.22)	0.002	3.87 (1.65–9.07)	0.002
40–64	24.13 (10.72–54.30)	<0.001	18.37 (8.13–41.49)	<0.001
≥65	71.69 (31.74–161.93)	<0.001	36.70 (16.00–84.17)	<0.001
Sex				
Female	Reference		Reference	
Male	1.58 (1.30–1.91)	<0.001	1.24 (1.02–1.51)	0.033
Hypertension	6.09 (5.03–7.38)	<0.001	1.47 (1.17–1.85)	0.001
Hyperlipidemia	4.56 (3.56–5.83)	<0.001	1.10 (0.84–1.45)	0.479
Chronic liver disease	2.63 (1.82–3.78)	<0.001	1.15 (0.79–1.66)	0.460
Chronic kidney disease	8.96 (5.34–15.03)	<0.001	2.38 (1.41–4.03)	0.001
Diabetes	7.86 (6.36–9.72)	<0.001	2.43 (1.91–3.09)	<0.001
COPD	4.59 (3.04–6.92)	<0.001	1.50 (0.99–2.29)	0.058
Ischemic heart disease	5.74 (4.34–7.59)	<0.001	1.27 (0.94–1.72)	0.114
Stroke	6.56 (4.72–9.12)	<0.001	1.51 (1.07–2.14)	0.020
Intracranial bleeding	2.93 (0.73–11.75)	0.130	0.73 (0.18–2.99)	0.664

PAOD, peripheral arterial disease; COPD, chronic obstructive pulmonary disease.

^†^ Adjusted for all variables.

**Table 4:** Subgroup of Cox proportional hazard model analysis.

	Non-psoriasis		Psoriasis		HR^†^ (95% C.I.)	*p* value
	*N*	No. of PAOD	*N*	No. of PAOD		
Age						
<20	2,571	NA	2,568	NA	0.20 (0.02–1.71)	0.141
20–39	5,912	NA	5,919	NA	1.20 (0.67–2.14)	0.543
40–64	5,313	95	5,316	126	1.34 (1.03–1.75)	0.031
≥65	1,863	77	1,856	97	1.25 (0.93–1.69)	0.140
*p* for interaction						<0.001
Sex						
Female	6,942	65	6,958	91	1.43 (1.04–1.97)	0.028
Male	8,717	133	8,701	158	1.19 (0.94–1.50)	0.140
*p* for interaction =						0.363
Hypertension						
No	13,642	118	13,652	153	1.33 (1.05–1.70)	0.019
Yes	2,017	80	2,007	96	1.16 (0.86–1.56)	0.336
*p* for interaction =						0.493
Hyperlipidemia						
No	14,710	160	14,712	210	1.31 (1.07–1.61)	0.010
Yes	949	38	947	39	1.06 (0.68–1.65)	0.810
*p* for interaction =						0.372
Chronic liver disease						
No	15,168	180	15,160	236	1.32 (1.09–1.61)	0.005
Yes	491	18	499	13	0.69 (0.34–1.42)	0.313
*p* for interaction =						0.080
Chronic kidney disease						
No	15,539	189	15,540	243	1.29 (1.07–1.57)	0.008
Yes	120	9	119	6	0.63 (0.22–1.78)	0.382
*p* for interaction =						0.162
Diabetes						
No	14,676	138	14,678	191	1.37 (1.10–1.70)	0.005
Yes	983	60	981	58	1.00 (0.70–1.44)	0.992
*p* for interaction =						0.140
Chronic obstructive pulmonary disease						
No	15,401	185	15,406	238	1.30 (1.07–1.57)	0.008
Yes	258	13	253	11	0.82 (0.36–1.86)	0.632
*p* for interaction =						0.292
Ischemic heart disease						
No	15,137	173	15,147	217	1.27 (1.04–1.55)	0.018
Yes	522	25	512	32	1.23 (0.73–2.08)	0.442
*p* for interaction =						0.865
Stroke						
No	15,328	177	15,329	231	1.30 (1.07–1.59)	0.008
Yes	331	21	330	18	0.85 (0.45–1.62)	0.624
*p* for interaction =						0.265
Intracranial bleeding						
No	15,625	NA	15,616	NA	1.28 (1.06–1.54)	0.010
Yes	34	NA	43	NA	NA (*N*A-NA)	NA
*p* for interaction =						0.961

NA, not applicable; PAOD, peripheral arterial disease.

^†^ Adjusted for all variables.

## Discussion

We enrolled 16,054 patients with newly diagnosed PSO from LHID 2000 from 2002 to 2017 in this study. We analyzed the association between PSO and the risk of PAOD using PSM. Our results showed that patients with PSO had a higher risk of acquiring PAOD than the non-PSO group. However, few studies have evaluated the risk association between PSO and PAOD. A case report described the co-occurrence of PSO with occlusive vascular disease and even gangrene on the lower limb in one patient (26). A case–control study demonstrated that PSO was an independent risk factor for certain cerebrovascular and peripheral vascular diseases (27). Because PAOD involves atherosclerosis of the lower extremities, here is discussed the association between PSO and atherosclerotic conditions other than PAOD.

PSO has been shown to increase the risk of CVD such as myocardial infarction, hypertension, valvular disease, and arrhythmia (28–34). PSO and atherosclerosis share certain common inflammatory pathways, such as endothelial dysfunction, cytokine dysregulation, platelet upregulation, and dyslipidemia (17, 35). The available body of evidence suggests the pathogenesis of vascular damage. On the contrary, the administration of TNF, IL-17A, and IL-12/23p40 inhibitors for the treatment of PSO was shown to lower the risk of CVD (19). PSO may have similar mechanisms that increase the risk of PAOD in CVDs.

We also found that the PSO group had an increased risk of PAOD with increasing age using the Cox proportional hazards model. A subgroup analysis indicated that patients aged 40–64 years had an increased risk of PAOD in the PSO group. A cross-sectional study showed that, especially for symptomatic PAOD, prevalence rates were higher in elderly groups between 50 and >70 years (36). Elderly patients with chronic obstructive pulmonary disease (COPD) also exhibited an increased risk of PAOD (37). The reasons for the increased prevalence with age may be associated with poor peripheral artery drainage and tissue loss. Previous studies showed that patients had comorbidities such as hypertension, hypercholesterolemia, chronic kidney disease, history of heart disease, COPD, and CVDs that also increase the risk of PAOD (38). In this study, we also showed that age was a risk factor for PAOD in PSO patients.

In addition to age, sex was a risk factor for PAOD. Male patients had a higher risk of PAOD than female patients. Sex-based differences in PAOD have been demonstrated in different studies. A study found a close relationship between visceral adiposity index and peripheral artery disease (PAD) in normal weight adults with hypertension among men but not among women (39). In contrast, a study of sex differences in critical limb ischemia, a serious form of PAD, indicated that women had a higher incidence of adjusted in-hospital mortality, bleeding complications, and discharge from facilities compared to men. This may be because women had a higher prevalence of obesity, hypertension, heart failure, and prior stroke than men (40). Similar findings indicated that women showed clinical signs of popliteal artery lesions at an older age than men (41). Factors affecting sex differences in PAOD include environmental, pathophysiologic, genetic and epigenetic, and treatment-related factors (42).

PSO may increase the risk of different diseases. The development of uveitis after PSO diagnosis has been reported (43). PSO increases the risk of organ-related comorbidities, such as CVD and renal and liver diseases (33). This may be due to predisposition genes and immunological mechanisms that PSO has in common with these diseases. For example, a review found that PSO and inflammatory bowel disease shared at least 11 common genes (44).

Although both diseases have common inflammatory pathways, the increased risk of PAOD with PSO may be due to common factors for both diseases, such as the overproduction of pro-inflammatory factors. PSO causes inflammation of the blood vessels, which is a critical step in initiating atherogenesis and the development of atherosclerosis (45). Pro-inflammatory cytokines and chemokines are involved in this complex process. For example, TNF-α is a pro-inflammatory factor that increases in patients with PSO and PAD (46). Another mechanism of vascular inflammation in patients with PSO involves activation of the inflammasome signaling pathway. In an ex vivo study, up-regulation of a series of inflammatory transcripts such as IL-1β, CXCL1, CCL3, CXCL-8, CXCL10, COX-2, ICAM-1, lymphotoxin beta, and VCAM-1 was found in endothelial cells of the brachial vein in patients with PSO. Blood levels of IL-6 were also elevated in these patients (47). CCL20, IL-17A, and IL-6 are critical pro-inflammatory factors that affect vascular health and contribute to endothelial inflammation (48). Elevation of these transcripts promotes atherosclerosis and increases CVD risk and can also increase the risk of PAOD in PSO patients.

Regardless of the actions of pro-inflammatory factors in blood vessels, serum markers can also be factors in PSO and PAOD. For example, galectin-3, which belongs to a member of galectins, a group of β-galactoside-binding proteins that exhibit biological functions such as cell–cell interaction, cell proliferation, and differentiation. Galectin-3 is postulated to be involved in the pathogenesis of a series of diseases, including the formation of psoriatic plaques and arthrosclerosis (49). Furthermore, a high level of galectin-3 was detected in the serum of PSO patients (24). Galectin-3 is also a potential cardiovascular inflammatory factor and mediator of arthrosclerosis (23). Galectin-3 and hs-CRP are involved in fibrosis in PAD (22). Based on the above evidence, galactin-3 may be involved in the pathogenesis in both PSO and arthrosclerosis and supported our finding that PSO increases the risk of PAOD.

This study still has certain limitations. First, LHID does not provide information about the severity or different forms of PSO, which may affect the risk and onset of PAOD. Second, the database does not provide data on whether the lesion is located in the lower extremity. However, this could directly influence the risk of PAOD. Third, the database did not provide information on smoking, alcohol consumption, physical activity, diet, inflammatory markers, disease progression, disease markers, body mass index (BMI), and other areas were not provided by the database; these factors are potential confounders in this study. Regardless, by using the propensity score matching in the age, sex, and comorbidities, we can reduce the potential heterogeneity of the two groups.

## Conclusion

In conclusion, our study demonstrates that having PSO significantly increases the risk of PAOD. Patients with PSO should be especially careful to prevent PAOD and other cardiovascular diseases. Additionally, age is a risk factor for PAOD, especially in patients with PSO 40–64 years of age.

## Data Availability

The original contributions presented in the study are included in the article/**Supplementary Material**, further inquiries can be directed to the corresponding author/s.
