# Supplementary material for: Association between psoriasis and peripheral artery occlusive disease: a population-based retrospective cohort study
Source: Front Cardiovasc Med. 2023 Jun 12;10:1136540. doi: 10.3389/fcvm.2023.1136540 (PMC10291070; doi:10.3389/fcvm.2023.1136540)
Supplement: Supplementary file 1 [file Datasheet1.pdf]

Supplementary table 1 Sensitivity analysis for risk of PAOD.

	Univariable		Multivariable†	
	HR (95% C.I.)	p value	HR (95% C.I.)	p value
Group				
Non-psoriasis	Reference		Reference	
Psoriasis	1.26 (1.04-1.51)	0.017	1.52 (1.23-1.88)	<0.001
Age				
<20	Reference		Reference	
20-39	3.94 (1.68-9.22)	0.002	3.81 (1.63-8.93)	0.002
40-64	24.13 (10.72-54.30)	<0.001	18.05 (7.98-40.83)	<0.001
≥65	71.69 (31.74-161.93)	<0.001	36.32 (15.78-83.59)	<0.001
Sex				
Female	Reference		Reference	
Male	1.58 (1.30-1.91)	<0.001	1.20 (0.99-1.46)	0.069
Hypertension	6.09 (5.03-7.38)	<0.001	1.48 (1.17-1.87)	0.001
Hyperlipidemia	4.56 (3.56-5.83)	<0.001	1.11 (0.84-1.46)	0.459
Chronic liver disease	2.63 (1.82-3.78)	<0.001	1.19 (0.82-1.72)	0.362
Chronic kidney disease	8.96 (5.34-15.03)	<0.001	2.42 (1.43-4.09)	0.001
Diabetes	7.86 (6.36-9.72)	<0.001	2.44 (1.91-3.11)	<0.001
COPD	4.59 (3.04-6.92)	<0.001	1.54 (1.01-2.34)	0.046
Ischemic heart disease	5.74 (4.34-7.59)	<0.001	1.28 (0.94-1.74)	0.112
Stroke	6.56 (4.72-9.12)	<0.001	1.46 (1.03-2.07)	0.036
Intracranial bleeding	2.93 (0.73-11.75)	0.130	0.73 (0.18-2.96)	0.656
Antiplatelet	3.97 (3.29-4.80)	<0.001	1.11 (0.89-1.38)	0.367
Anticoagulant	3.09 (1.93-4.95)	<0.001	0.85 (0.53-1.38)	0.511
Methotrexate	1.08 (0.73-1.60)	0.695	0.86 (0.57-1.31)	0.489
Sulfasalazine	0.86 (0.43-1.74)	0.681	0.77 (0.37-1.62)	0.489
Corticosteroids	0.77 (0.60-1.00)	0.047	0.48 (0.36-0.65)	<0.001

PAOD: Peripheral arterial disease
COPD: Chronic obstructive pulmonary disease.
†Adjusted for all variables.
